# Spatially Selective Imaging in Color: What You See is What You Want

**DOI:** 10.1002/advs.202411537

**Published:** 2024-12-27

**Authors:** John You En Chan, Akshaya Rajesh, Xiaoyu Lin, Hao Wang, Hongtao Wang, Xiaoyan Zhou, Cheng‐Wei Qiu, Joel K.W. Yang

**Affiliations:** ^1^ Engineering Product Development Singapore University of Technology and Design Singapore 487372 Singapore; ^2^ School of Instrumentation and Optoelectronic Engineering Beihang University Beijing 100191 China; ^3^ Department of Electrical and Computer Engineering National University of Singapore Singapore 117583 Singapore

**Keywords:** fresnel lenses, imaging, multiplexing, structural colors, two photon polymerization

## Abstract

Spatially selective imaging (SSI) involves sampling a group of pixels from different positions on an encoded object to display a decoded image. Here, SSI is achieved by using off‐axis cylindrical Fresnel lens arrays to decode multiple images from an encoded print of structural color pixels. Each image is optically retrieved by separately placing different “keys” (arrays of lenses in different pseudorandom configurations) over the same encoded print, and then each image is digitally reconstructed for visualization. In addition, a detailed analysis is presented on the designed lenses and pixels, which are all fabricated by two‐photon polymerization lithography. Though Fresnel lenses are susceptible to chromatic aberrations, the results show that they can be used for imaging pixels that produce a wide range of colors at low numerical aperture (*NA* ≈0.2). The images have acceptable color contrast, corresponding to a simulated normalized modulation transfer function (*MTF*) of ≈0.6 averaged across wavelengths in the visible spectrum. This work can find applications in optical information security devices.

## Graphical abstract

When an encoded structural color print is combined with an off‐axis Fresnel lens array, a decoded image is displayed that appears different from the print. The decoded image is formed by selected color pixels across pseudorandom spatial positions on the print. As multiple decoded images can be displayed from the same print, this work has potential applications in optical information security devices.

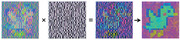

## 1 Introduction

Conventional imaging is often described as a “what‐you‐see‐is‐what‐you‐get” technique, in which the visible information of an entire object is directly captured by one lens and displayed to an observer. In contrast, spatially selective imaging (SSI) can be described as a “what‐you‐see‐is‐what‐you‐want” technique,^[^ ^1^ ^]^ in which the visible information from some parts of the object is sampled by a lens array to display an image that appears different from the entire object. Hence, SSI not only unveils a creative way of seeing the world, but also enables multiple sets of information to be decoded from a strategically encoded object.

**Figure 1:**
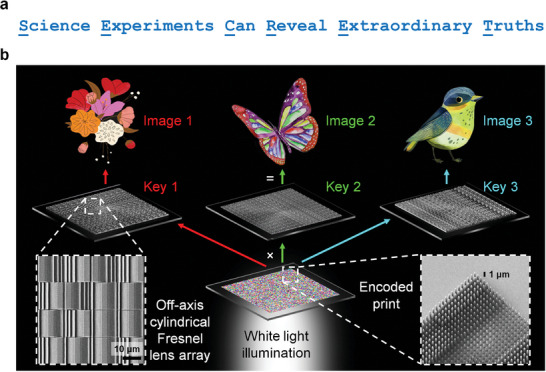
The concept of spatially selective imaging (SSI). a) Text analogy of SSI. By combining the first letter (capitalized and underlined) of each word in the sentence from left to right, a message that spells “SECRET” is obtained. b) Schematic of an encoded print comprising structural color pixels (nanopillars), separately combined with three different “keys” (off‐axis cylindrical Fresnel lens arrays) to reveal three different images (e.g. flower, butterfly, bird) under ordinary white light illumination. Scanning electron microscope images of an off‐axis cylindrical Fresnel lens array and the encoded print are shown in the insets.

This work is inspired by the idea of hiding a message within a larger piece of text. For example, a message can be hidden in chosen letters that are distributed across different words in a sentence (**Figure** **1**a). The message is then retrieved by extracting the letters at selected positions from each word. We extend this idea to SSI, in which the message is an image, and the letters are pixels. Whilst hidden messages are decoded by mathematical algorithms, hidden images can be decoded by optical components that offer an extra physical layer of security. To retrieve one of the hidden images, an off‐axis cylindrical Fresnel lens array (also called a “key”) is used to sample a set of structural color pixels at selected spatial positions on an encoded print. When different keys are separately used to select different sets of pixels on the same encoded print, multiple hidden images are retrieved (Figure 1b). This concept of retrieving multiple images from a single encoded print is also found in holograms^[^ ^2^ ^]^ that exploit light through several degrees of freedom,^[^ ^3^, ^4^ ^]^ such as amplitude,^[^ ^5^ ^]^ phase,^[^ ^6^ ^]^ wavelength,^[^ ^7^ ^]^ polarization,^[^ ^8^, ^9^ ^]^ and orbital angular momentum.^[^ ^10^, ^11^, ^12^ ^]^ However, these holograms generally require coherent or structured light illumination, whereas SSI requires only ordinary white light illumination with a caveat: that the off‐axis cylindrical Fresnel lens array must be aligned accurately and precisely to the encoded print, analogous to the workings of a mechanical key and lock.

SSI is a variant of integral imaging,^[^ ^13^, ^14^, ^15^, ^16^, ^17^ ^]^ where an on‐axis lens array is generally used to select different sets of pixels that are displayed according to the observation angle. However, the on‐axis lens array limits the arbitrary selection of pixel positions that can provide greater design flexibility and complexity. Our approach uses off‐axis cylindrical Fresnel lens arrays to achieve arbitrarily selected pixel positions for a fixed observation angle = 0° (with respect to the optical axis). Instead of varying the observation angle, different sets of pixels are selected and displayed by off‐axis cylindrical Fresnel lens arrays with varying configurations. Compared to traditional refractive lenses, Fresnel lenses have reduced thicknesses (≈2*λ*) due to their modulo‐2π phase profiles that enable shorter fabrication times for compact and lighter devices.^[^ ^18^ ^]^ On the other hand, metalenses have subwavelength thicknesses (≈*λ*/2) and even demonstrated remarkable functionalities such as polarization sensitivity^[^ ^19^ ^]^ and tunable focusing,^[^ ^20^ ^]^ but Fresnel lenses are sufficient for SSI in which only the image intensity is concerned.^[^ ^21^, ^22^ ^]^ Due to their large chromatic aberrations,^[^ ^23^, ^24^ ^]^ Fresnel lenses have been used mostly in non‐imaging applications^[^ ^25^ ^]^ (e.g. solar concentrators), and less commonly in color imaging.^[^ ^13^, ^17^ ^]^ Chromatic aberrations in Fresnel lenses are primarily caused by their wavelength‐dependent phase profiles. When a Fresnel lens is designed with a fixed phase profile for only one operation wavelength (*λ*_o_ = 550 nm), a broad range of wavelengths (*λ* = 380–780 nm) gets focused to varying focal spot positions.^[^ ^26^ ^]^ Despite this phenomenon, we show that Fresnel lenses uncorrected for chromatic aberrations can still be used for color imaging through meticulous design.

Our design requires lenses with low numerical aperture (*NA* ≈0.2) to display the expected colors of the pixels. Specifically, the color at *NA* = 0.20 is indistinguishable by an average human observer from the color at *NA* = 0.10 to 0.30, within a 3‐step MacAdam ellipse on the CIE 1976 *u’*–*v’* diagram. This ellipse quantifies an acceptable threshold of chromaticity variations in all directions from the chromaticity coordinates at the center of the ellipse.^[^ ^27^ ^]^ At high *NA* → 1, the color becomes desaturated compared to the color at *NA* = 0.20. Though the *NA* of the lenses varies with wavelength due to chromatic aberrations, the *NA* remains within the acceptable range from 0.10 to 0.30 for wavelengths in the visible spectrum. We fabricate the lenses and the pixels by two‐photon polymerization lithography as it is capable of rapid prototyping customized 3D photonic structures with sub‐micrometer resolution.^[^ ^28^, ^29^, ^30^ ^]^ To demonstrate SSI, we separately combine an encoded print (comprising an array of sub‐pixels) with different off‐axis cylindrical Fresnel lens arrays to display multiple decoded images. Subsequently, we digitally reconstruct these images for visualization. The images have acceptable color contrast as the simulated normalized modulation transfer function (*MTF*) is ≈0.6 averaged across wavelengths in the visible spectrum. This *MTF* value corresponds to the horizontal spatial frequency of the sub‐pixels (0.20 cycles µm^−1^), which is below the lowest cutoff spatial frequency of the lenses (0.62 cycles µm^−1^) for wavelengths in the visible spectrum. Our successful demonstration of SSI signifies potential applications in optical information security devices.

## 2 Results

### 2.1 Design of the Structural Color Pixels

**Figure 2:**
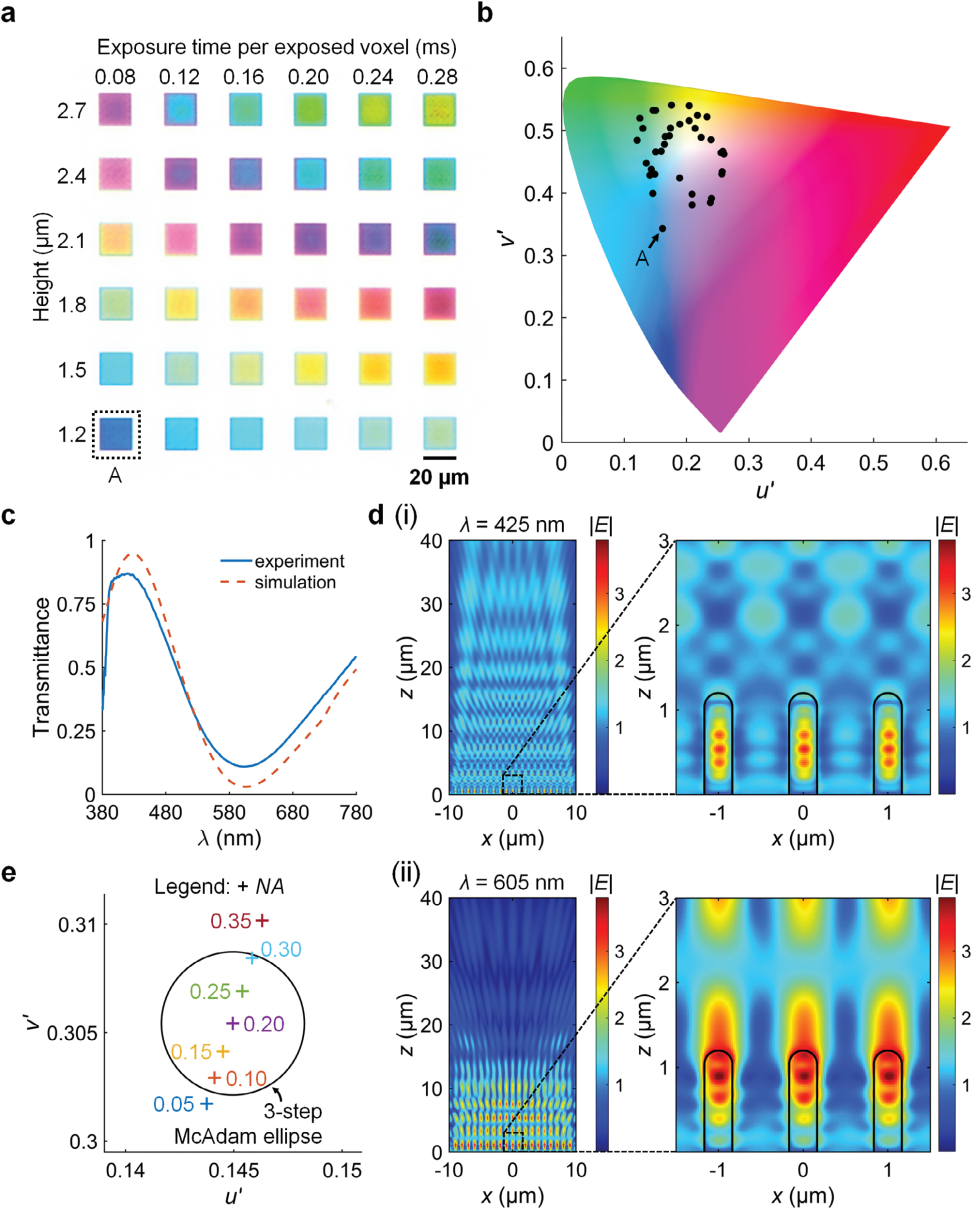
Design of the structural color pixels. a) Optical image of a fabricated color palette. The image was captured in transmission mode by a microscope objective with numerical aperture *NA* = 0.20. Pixel A was selected for further analysis. b) CIE 1976 *u’*–*v’* diagram that shows the chromaticity coordinates of all pixels in the fabricated color palette. The chromaticity coordinates were plotted for the D65 standard illuminant and CIE 1931 2° standard observer. c) Simulated (red dashed line) and experimentally measured (blue solid line) transmittance spectra of Pixel A. d) Simulated electric field magnitude (|*E*|) distributions of Pixel A on the *x*–*z* plane at *y* = 0 µm for (i) *λ* = 425 nm (peak wavelength) and (ii) *λ* = 605 nm (trough wavelength). The enlarged‐view electric field magnitude distributions are shown in the insets, where the thick solid lines outline the nanopillars of Pixel A. e) Simulated chromaticity coordinates of Pixel A for varying *NA*. A 3‐step MacAdam ellipse is shown with its center coordinates (*u_c_’* = 0.1450, *v_c_’* = 0.3054) corresponding to the color observed at *NA* = 0.20.

Initially, we fabricated a palette of structural color pixels and observed the pixels through a microscope objective with numerical aperture *NA* = 0.20 (**Figure** **2**a). Each pixel comprised a pseudo‐periodic array of nanopillars (period = 1 µm) with varying heights (1.2–2.7 µm), and diameters (0.3–0.4 µm) that were indirectly controlled by the laser exposure time per exposed voxel (0.08–0.28 ms). These nanopillars are known to produce a wide range of colors^[^ ^31^ ^]^ and have been used to create many sophisticated color prints.^[^ ^7^, ^16^, ^32^, ^33^ ^]^ However, the color dependence of these nanopillars on the *NA* of the lens (or microscope objective) has not been scrutinized. In this section, we provide deeper insights into this color dependence. For ease of visualization and spectra measurement, we fabricated each pixel in the palette to have 20 µm width, rather than the 5 µm width used in the encoded print (see Demonstration of Spatially Selective Imaging (SSI) Section). We previously demonstrated that changes in pixel width at the micrometer scale do not have a noticeable effect on the pixel color observed at long distances beyond the millimeter scale.^[^ ^16^ ^]^ Next, we calculated the chromaticity coordinates of the pixels based on their transmittance spectra, the theoretical D65 standard illuminant, and the CIE 1931 2° standard observer (Note S1, Supporting Information). Instead of plotting the chromaticity coordinates on the widely used CIE 1931 *x*–*y* diagram, we plotted the chromaticity coordinates on the CIE 1976 *u’*–*v’* diagram (Figure 2b) because it more accurately represents the perceptual uniformity of color‐normal human observers. Hence, on the CIE 1976 *u’*–*v’* diagram, chromaticity differences with similar magnitudes are likely to correspond to similar amounts of perceived change in color.

From the palette, we selected Pixel A (height = 1.2 µm, exposure time per exposed voxel = 0.28 ms) to analyze its color. In our simulation (see Experimental Section), we modeled Pixel A as an array of nanopillars (cylinders capped with hemispheres). We set the refractive index of the nanopillars according to the Cauchy parameters of IP‐Dip resin exposed by two‐photon polymerization lithography (Figure S1, Supporting Information). The Cauchy parameters are reported by Dottermusch et. al.^[^ ^34^ ^]^ The transmittance spectrum of Pixel A obtained from simulation showed reasonable agreement with experiment (Figure 2c). However, fabrication imperfections in Pixel A could have caused a reduced contrast in the experiment spectrum compared to the simulation spectrum. We quantified the contrast using Equation (1):

(1) $$C=\frac{T_{max}-T_{min}}{T_{max}+T_{min}}\;$$

where *T_max_* and *T_min_* represent the maximum and minimum transmittance of the spectrum respectively. *C* = 0.77 in the experiment spectrum, and *C* = 0.93 in the simulation spectrum. The maximum contrast of *C* = 1 was not attained in the simulation spectrum due to reflection at the substrate‐nanopillars interface and scattering by the nanopillars. Moreover, the reduced contrast in the experiment spectrum can be attributed to non‐normal incident angles of light, whereas the simulation spectrum considered only normally incident light. To understand the color mechanism of Pixel A, we also simulated its electric field magnitude distribution on the *x*–*z* plane for the peak wavelength *λ* = 425 nm and the trough wavelength *λ* = 605 nm. Within −10 µm < *x* < 10 µm (the width of Pixel A), the electric field magnitude distribution from *z* > 20 µm revealed constructive interference for *λ* = 425 nm and destructive interference for *λ* = 605 nm (Figure 2d), results that are consistent with the simulation spectrum. However, Figure 2d alone does not reveal the transmitted wave propagation direction that, we propose, is responsible for the color dependence of Pixel A on the *NA* of the lens. As the *NA* describes the lens acceptance cone angle, only specific wavelengths of transmitted light that propagate within this cone are collected by the lens and contribute to color. To obtain the propagation angle of the transmitted wave, we simulated a near to far field transformation of the electric field distribution on the *x*–*y* plane. The electric field intensity distribution in the far field was calculated on a hemispherical surface located at z ≈1 m. Viewed from above, this hemispherical surface is a polar image that represents the propagation angles of transmitted light. The distribution for *λ* = 425 nm revealed a distinct peak at the center, whereas the distribution for *λ* = 605 nm revealed several peaks away from the center (Figure S2, Supporting Information). This simulation result is consistent with the experimental result reported by Nawrot et. al.^[^ ^31^ ^]^ Hence, both results support the proposition that the color of Pixel A (and other pixels in the palette) depends on the *NA* of the lens.

To verify this proposition, we simulated the color of Pixel A for *NA* = 0.05 to *NA* = 0.95 in steps of 0.05, and then plotted the corresponding chromaticity coordinates on the CIE 1976 *u’*–*v’* diagram. On this diagram, we also plotted a 3‐step MacAdam ellipse with center coordinates (*u_c_’* = 0.1450, *v_c_’* = 0.3054) corresponding to the color observed at *NA* = 0.20. This ellipse is used to quantify an acceptable threshold for chromaticity changes in Pixel A. The equation of this ellipse adopts the definition from CIE TN 001:2014, as shown in Equation (2):

(2) $${\left(u^{\prime }-{u_{c}}^{\prime }\right)}^{2}+{\left(v^{\prime }-{v_{c}}^{\prime }\right)}^{2}={\left(0.0011\;m\right)}^{2}$$

where *m* is the number of steps. We set *m* = 3, so that colors with chromaticity coordinates that lie outside this ellipse are distinguishable from the color at (*u_c_’*,*v_c_’*) by 99.7% of the population of color‐normal human observers. For Pixel A, only the chromaticity coordinates from *NA* = 0.10 to 0.30 laid inside the ellipse (Figure 2e). As the *NA* increased, the chromaticity coordinates of Pixel A shifted toward the chromaticity coordinates of the D65 standard illuminant white point (*u_w_’* = 0.1978, *v_w_’* = 0.4683) (Table S1, Supporting Information). This trend suggests that Pixel A transmits all wavelengths of visible light, but only specific wavelengths contribute to color if they are collected by the imaging lens. In other words, at low *NA* → 0, Pixel A appears blue as the propagation angle of transmitted light for *λ* = 425 nm is within the lens acceptance cone angle, but not for *λ* = 605 nm. At high *NA* → 1, Pixel A would appear desaturated as the propagation angles of visible spectrum wavelengths are within the lens acceptance cone angle (Figure S3, Supporting Information). Indeed, for pixel A, we observed the color difference between low *NA* and high *NA* (Figure S4, Supporting Information). Hence, these structural color pixels are appropriate for low *NA* imaging, which would be achieved by our designed Fresnel lenses.

### 2.2 Design of the Fresnel Lenses

**Figure 3:**
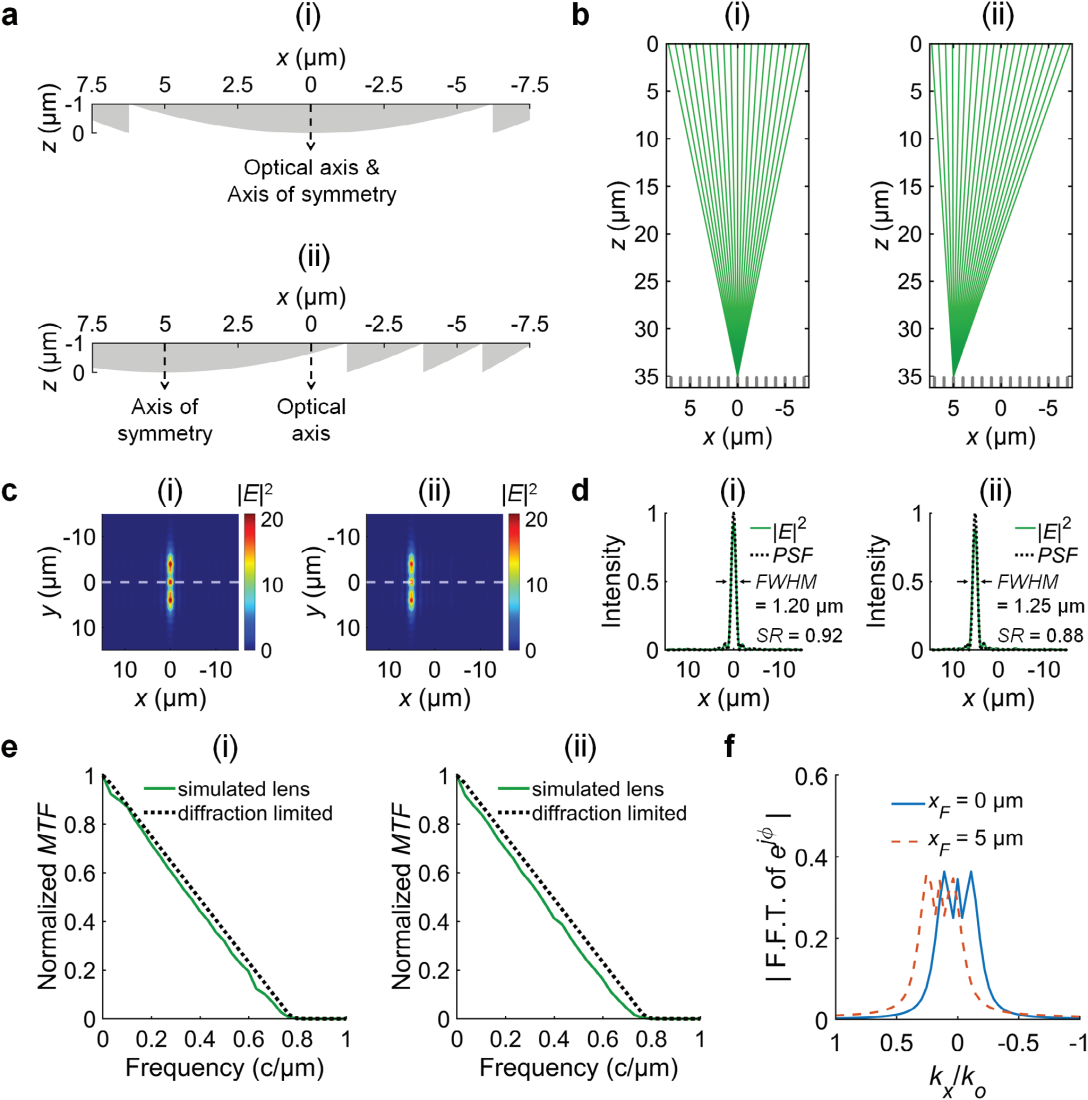
Design of the Fresnel lenses. All simulation results shown here are for operation wavelength *λ* = 550 nm. a) Thickness profiles of (i) the on‐axis lens (*x_F_* = 0 µm, *z_F_* = 35 µm), and (ii) the off‐axis lens (*x_F_* = 5 µm, *z_F_* = 35 µm). *x_F_* and *z_F_* denote the coordinates of the focal point. For illustration, the lenses have been rotated upside down so that they face toward the color pixels when imaging. b) Simulated raytracing diagrams of each lens imaging a pixel (represented by an array of nanopillars). c) Simulated electric field intensity (|*E*|^2^) distributions on the *x*–*y* plane at *z* = 35 µm. The electric field intensity profiles were measured along the *x*‐direction at *y* = 0 µm, indicated by the white dashed lines. d) Graphs of the electric field intensity profile (green solid curve), and the point spread function (*PSF*) (black dotted curve) of a square aperture (15 µm × 15 µm). In each graph, the electric field intensity profile was normalized to yield the same area under the curve as the *PSF*. The *SR* refers to the Strehl ratio, and the *FWHM* refers to the full‐width‐at‐half‐maximum of the electric field intensity profile. e) Normalized modulation transfer function (*MTF*) graphs of the simulated lenses and their diffraction‐limited cases. f) Fourier magnitude spectrum of *e^jϕ^* for the on‐axis lens (*x_F_* = 0 µm) and the off‐axis lens (*x_F_* = 5 µm), where *ϕ* is the hyperbolic phase profile, and F.F.T. refers to the Fast Fourier Transform.

Since we found the structural color pixels to be appropriate for low *NA* imaging, we designed the Fresnel lenses to have *NA* ≈0.2, close to the *NA* of the microscope objective that was used to image the pixels (Figure 2a). Based on geometry, the *NA* of each lens is given by Equation (3):

(3) $$NA=\sin\left(\frac{1}{2}\left(\tan^{-1}\left(\frac{\frac{D}{2}+x_{F}}{z_{F}}\right)+\tan^{-1}\left(\frac{\frac{D}{2}-x_{F}}{z_{F}}\right)\right)\right)$$

where *D* is the side length of the lens, *x_F_* and *z_F_* are the coordinates of the focal point. Each lens was designed with a modulo‐2π hyperbolic phase profile to allow constructive interference of normally incident light waves at the focal point. The phase profile is given by Equation (4):

(4) $$\phi =\mathrm{mod}\left(-k_{o}\left(\sqrt{{\left(x-x_{F}\right)}^{2}+{z_{F}}^{2}}-z_{F}\right),2\pi \right)$$

where *k_o_* = 2π/*λ_o_* is the wavenumber, *λ_o_* is the designed operation wavelength. The corresponding lens thickness profile is given by Equation (5):

(5) $$t=\frac{\phi }{k_{o}\left(n-1\right)}$$

where *n* is the lens refractive index. In our design, we set *n* = 1.55 and *λ_o_* = 550 nm. We also set *z_F_* = 35 µm and *x_F_* = [−5, 0, 5] µm to obtain three lens profiles that constitute the off‐axis Frensel lens array. Each lens had a maximum thickness of 1 µm, and equal side lengths of *D* = 15 µm. As the designed phase profile does not vary along the *y*‐direction, the lens is cylindrical. Note that a lens designed for *x_F_* = 0 is on‐axis, but *x_F_* ≠ 0 is off‐axis. Though both on‐axis and off‐axis lenses were later combined into an array for spatially selective imaging (SSI), for convenience we will refer to this array as an “off‐axis cylindrical Fresnel lens array” as it does not detract from the understanding of SSI. The (i) on‐axis and (ii) off‐axis Fresnel lenses have different structures, as shown in their thickness profiles (**Figure** **3**a). In (i), the optical axis and the axis of symmetry coincide. However, in (ii), the axis of symmetry is displaced from the optical axis along the *x*‐direction. Consequently, the on‐axis and off‐axis Fresnel lenses have focal points at different positions along the *x*‐direction, as shown in their simulated raytracing diagrams for *λ* = 550 nm (Figure 3b). Because these lenses were designed for low *NA* imaging of structural color pixels, the color of a pixel can be observed through a lens only if the pixel is placed at the focal point.

To evaluate the imaging performance of the lenses, we simulated their electric field intensity (magnitude squared) distributions on the *x*–*y* plane at *z* = 35 µm for *λ* = 550 nm (Figure 3c). These distributions revealed elongated focal spots, as the lenses were cylindrical along the *y*‐direction. In addition, the lenses had near diffraction‐limited resolution for *λ* = 550 nm, as the Strehl ratio (*SR*) was (i) 0.92 and (ii) 0.88 respectively. To calculate the *SR* of each lens, we examined its electric field intensity profile along the *x*‐direction at *y* = 0 µm. We then normalized the profile to yield the same area under the curve (from −7.5 µm < *x* < 7.5 µm) as the shifted point spread function (*PSF*) of a square aperture. This *PSF* is given by Equation (6):

(6) $$PSF=\sin c^{2}\;\left(\frac{\pi D\left(x-x_{F}\right)}{\lambda z_{F}}\right)\sin c^{2}\left(\frac{\pi Dy}{\lambda z_{F}}\right)$$

where sinc(*x*) = sin(*x*)/*x*. Note that this *PSF* describes the intensity distribution of an object point on the image plane, if the object point is imaged by a diffraction‐limited lens with no aberrations. For comparison, we plotted the diffraction‐limited *PSF* and the normalized electric field intensity profile of each lens on the same graph (Figure 3d). The calculated *SR* was the ratio of the maximum intensity in the normalized electric field intensity profile to the maximum value of unity in the *PSF*.

We also simulated the modulation transfer function (*MTF*) of the lenses and verified their near diffraction‐limited resolution for *λ* = 550 nm (Figure 3e). Residual differences between the simulated lens *MTF* and the diffraction‐limited *MTF* can be attributed to on‐axis (spherical) and off‐axis (coma) aberrations. To calculate the *MTF* of each lens, we numerically integrated its electric field intensity distribution on the *x*–*y* plane along the *y*‐direction. This numerical integration resulted in a line spread function (LSF) along the *x*‐direction. We then performed a Fast Fourier Transform on the LSF to obtain the optical transfer function (OTF). The magnitude of the OTF gave the *MTF*. We then normalized the *MTF* to yield a value of 1 at zero spatial frequency. As the spatial frequency increased, the normalized *MTF* value decreased until it became effectively 0 beyond a cutoff spatial frequency *f_c_*, at which the lens cannot resolve higher spatial frequency components. We calculated *f_c_* to be around 0.87 cycles µm^−1^, corresponding to a full‐width‐at‐half‐maximum (*FWHM*) value of 1.14 µm in the *PSF*. On the other hand, the *FWHM* value was (i) 1.20 µm and (ii) 1.25 µm respectively in the simulated electric field intensity profile of each lens.

Using these *FWHM* values, we calculated the efficiency of the lenses to be (i) 0.81 and (ii) 0.76 respectively. We defined efficiency as the optical power collected within a rectangular area (6*FWHM × 15 µm), divided by the total incident power within the lens area (15 µm × 15 µm).^[^ ^35^, ^36^ ^]^ The reduced efficiency in the off‐axis lens can be explained by its focal spot angle with respect to the *z*‐direction in the *x*–*z* plane (Figure S5, Supporting Information), as the optical power per unit area is a cosine‐dependent function of this angle. Moreover, the focal spot angle is related to the symmetry in the Fourier magnitude spectrum of *e^jϕ^* (Figure 3f).^[^ ^37^ ^]^ In the on‐axis lens, the spectrum was symmetric about *k_x_*/*k_o_* = 0, so the balanced distribution of positive and negative *k_x_/k_o_* components produced a focal spot angle of zero (i.e. the focal spot is not slanted). However, in the off‐axis lens, the asymmetric spectrum caused an imbalanced distribution of *k_x_/k_o_* components, and this imbalanced distribution produced a focal spot angle greater than zero (i.e. the focal spot is slanted). Despite the reduced efficiency in the off‐axis lens, it still maintained a high relative efficiency of ≈94% to the on‐axis lens. Hence, these lenses can be combined into an array for SSI with slight spatial variations in image brightness.

To investigate chromatic aberrations in the designed Fresnel lenses, we did further raytracing simulations for wavelengths at the lower and upper limits of the visible spectrum (*λ* = 380 nm and *λ* = 780 nm) (Figure S6, Supporting Information). The different wavelengths shifted the position of the focal point and produced a defocused spot on the plane *z* = 35 µm. Though the *NA* of the lenses were different for *λ* = 380 nm and *λ* = 780 nm, the *NA* remained within the acceptable range from 0.10 to 0.30, determined by the 3‐step MacAdam ellipse of Pixel A (see Design of the Structural Color Pixels Section). Hence, the expected colors of the pixels can be observed through the lenses. To further investigate chromatic aberrations, we simulated the electric field intensity distribution of the lenses on the *x*–*y* plane at *z* = 35 µm for *λ* = 380 nm and *λ* = 780 nm (Figure S7, Supporting Information). In each lens and for each wavelength, we examined the electric field intensity profile along the *x*‐direction at *y* = 0 µm and calculated the strehl ratio. For *λ* = [380, 780] nm respectively, the *SR* = [0.42, 0.59] in (i), and the *SR* = [0.26, 0.27] in (ii). These *SR* values indicate that the lenses have reduced resolution at the wavelength limits of the visible spectrum than at the designed operation wavelength *λ_o_ =* 550 nm. To verify that the lenses have sufficient resolution for broadband imaging, we also simulated the normalized *MTF* of the lenses for wavelengths across the visible spectrum (Figure S8, Supporting Information). For *λ* = [380, 780] nm, the cutoff spatial frequencies were *f_c_* = [1.27, 0.62] cycles µm^−1^, respectively. The lowest cutoff spatial frequency (0.62 cycles µm^−1^) imposes an upper limit on the spatial frequency of the pixels. In the encoded print used for SSI, we designed each sub‐pixel to have 5 µm width, which corresponds to a spatial frequency of 0.20 cycles µm^−1^ and a simulated normalized *MTF* ≈0.6 averaged across wavelengths in the visible spectrum. Hence, the lenses are expected to resolve the pixels and produce images with acceptable color contrast.

### 2.3 Demonstration of Spatially Selective Imaging (SSI)

**Figure 4:**
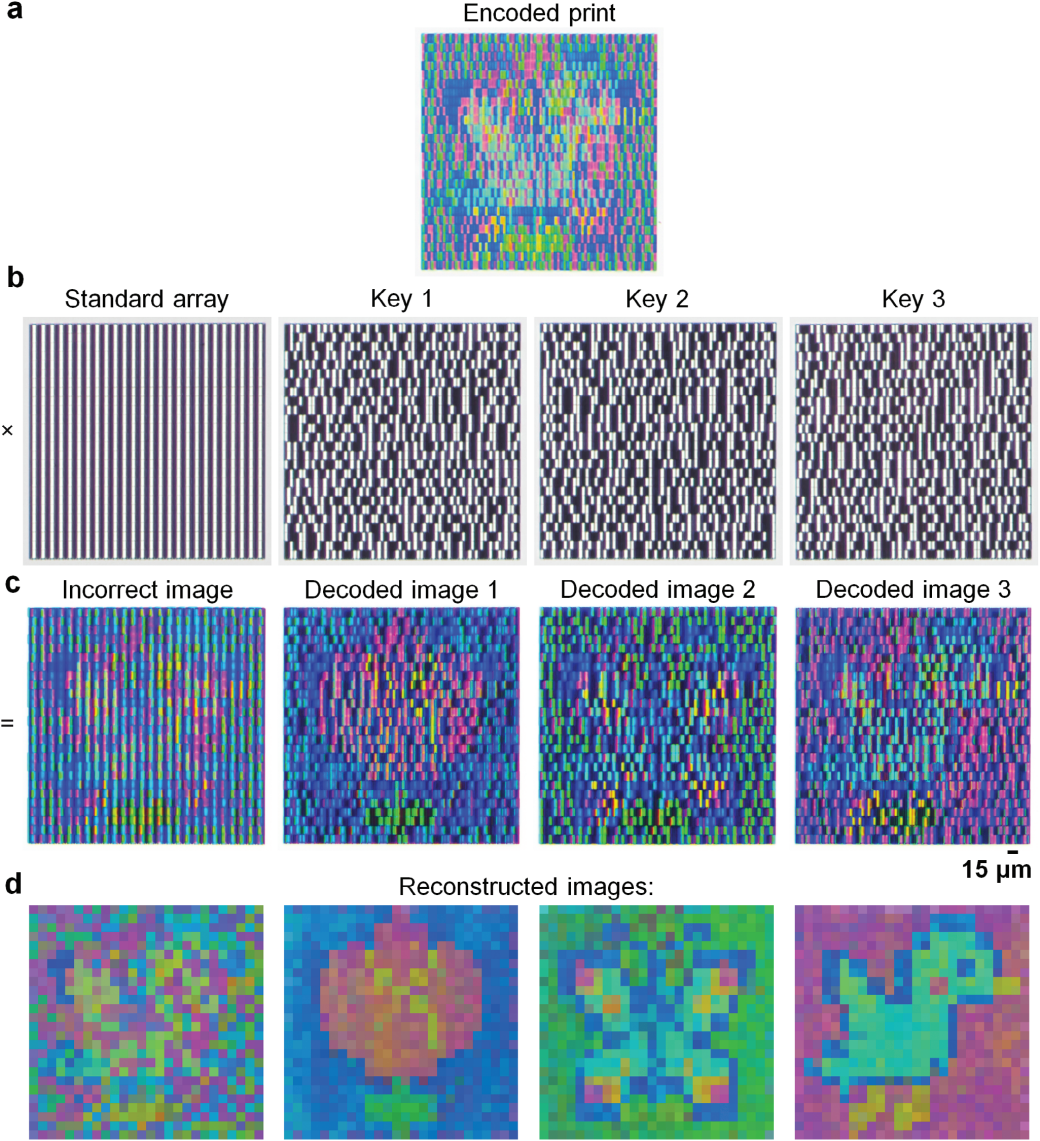
Experimental demonstration of spatially selective imaging. a) Optical image of the encoded print. b) Optical images of four different cylindrical Fresnel lens arrays: a standard array, and three “keys”. The standard array comprised only on‐axis lenses, whereas each key comprised both off‐axis and on‐axis lenses in a different pseudo‐random configuration. c) Optical images of the encoded print displayed through the four different cylindrical Fresnel lens arrays. When the standard array was aligned to the encoded print, an incorrect image with a pseudo‐random distribution of colors was displayed. When the keys were separately aligned to the encoded print, the decoded images of a pixelated flower, butterfly, and bird were displayed. d) Digitally reconstructed images of the optical images in c).

Based on the designs of the structural color pixels and Fresnel lenses, we fabricated two samples (see Experimental Section). The first sample was an encoded print (**Figure** **4**a) that comprised an array of 78 × 26 rectangular sub‐pixels in the horizontal × vertical dimensions. The sub‐pixels were multiplexed from 3 different input images and pseudo‐randomly assigned to different positions under 26 × 26 lenses, in which each lens (15 µm × 15 µm) covered 3 sub‐pixels: 1 sub‐pixel (5 µm × 15 µm) per input image. This pseudo‐random assignment of sub‐pixels is analogous to a block transposition cipher that rearranges the order of letters in fixed‐length groups of text. The second sample comprised a standard lens array and 3 different “keys”. The standard lens array comprised only on‐axis (*x_F_* = 0 µm) lenses, whereas each key comprised both on‐axis and off‐axis (*x_F_* = [−5, 5] µm) lenses in different pseudorandom configurations (Figure 4b). The configuration of lenses in each key was designed to select the correct set of sub‐pixels corresponding to each input image.

To demonstrate SSI, we gently aligned the second sample on top of the first sample, such that the lenses faced toward the pixels. Both samples were separated by a spacer layer (adhesive tape) of around 35 µm thickness corresponding to the designed value of *z_F_*. A broadband white light source was used to illuminate the first sample at normal incidence, and the image displayed through the second sample was observed in transmission mode using an optical microscope. Different images were displayed as the standard lens array and the keys were separately aligned to the encoded print (Figure 4c). The alignment was achieved by manual adjustments and visual inspection. When the standard lens array was aligned to the encoded print, an incorrect image with a pseudo‐random distribution of colors was displayed. Instead, when each key was aligned to the encoded print, the decoded images of a pixelated flower, butterfly, and bird, were displayed. Each key selected the correct set of sub‐pixels due to the focusing effect of individual lenses (Figure S9, Supporting Information). Notice that, in Figure 4b, the lenses produced bright areas at the positions of the focal spots, and darks areas where there are no focal spots. These bright and dark areas enabled SSI, as sub‐pixels that overlapped with the bright (dark) areas were transmitted (blocked). Though the dark areas reduced the visibility of the decoded images, the images can still be perceived in Figure 4c.

To enhance the visibility, we used a simple algorithm that extracted the colors from bright areas in the decoded images, and then combined these colors to create digitally reconstructed images without the dark areas (Figure 4d). The reconstructed images appeared pixelated as they were formed by a small number of sub‐pixels (26 × 26 per image), which can be increased at the cost of longer fabrication times. Moreover, the reconstructed images appeared noisy due to variations in lens efficiency and non‐uniform colors caused by fabrication imperfections. Despite their pixelated and noisy appearance, the reconstructed images revealed almost no visual distortion or crosstalk as each lens array was accurately aligned to the encoded print. In addition, the reconstructed images revealed acceptable color contrast as the simulated normalized *MTF* was ≈0.6 averaged across wavelengths in the visible spectrum (Figure S8c, Supporting Information). This *MTF* value corresponded to the spatial frequency of the sub‐pixels (0.20 cycles µm^−1^), which was below the lowest cutoff spatial frequency of the lenses (0.62 cycles µm^−1^) for wavelengths in the visible spectrum. Though we only demonstrated 3 decoded images from an encoded print through SSI, it is possible to decode a greater number of images *I* given by Equation (7):

(7) $$I=\mathrm{floor}\left(\frac{D}{W}\right)\leq \frac{D}{\lambda_{o}/\left(2NA\right)}$$

where *D* is the side length of each lens, *W* is the width of each sub‐pixel, *NA* is the lens numerical aperture, and *λ_o_* is the designed operation wavelength. The maximum *I* is determined by the diffraction‐limited resolution of the lens (*λ_o_*/2*NA*). However, due to lens aberrations, the actual *I* is less than the maximum *I*. Though *I* can be maximized by increasing *D*, larger lenses will reduce the resolution of the images and cause the images to appear more pixelated. In practice, *D* < 50 µm is required to display sufficiently high‐resolution images observed by the human eye.

## 3 Discussion

Spatially selective imaging (SSI) is a “what‐you‐see‐is‐what‐you‐want” technique. By using off‐axis cylindrical Fresnel lens arrays to sample selected structural color pixels in an encoded print, multiple images that appear different from the encoded print itself are displayed. This effect would not be achieved in conventional imaging, a “what‐you‐see‐is‐what‐you‐get” technique that displays an image of the entire object. Our successful demonstration of SSI was achieved by meticulous design of the structural color pixels and Fresnel lenses.

In the design of the structural color pixels, we found that the pixels were appropriate for low numerical aperture (*NA*) imaging, as the pixels transmitted different wavelengths of light to varying propagation angles. For a selected pixel, we found that the simulated color change from *NA* = 0.10 to *NA* = 0.30 was indistinguishable by an average human observer within a 3‐step MacAdam ellipse centered at *NA* = 0.20 on the CIE 1976 *u’*–*v’* diagram. As the *NA* → 1, the color of that pixel became desaturated, because its chromaticity coordinates approached the white point of the theoretical D65 standard illuminant used in our analysis. The design of the structural color pixels influenced the design of the Fresnel lenses. To display the expected colors of the pixels, we designed the lenses with *NA* ≈0.2. Though the lenses suffered from chromatic aberrations that resulted in different *NA* for different wavelengths, the *NA* remained within the acceptable range from 0.10 to 0.30 for wavelengths in the visible spectrum (*λ* = 380 nm to *λ* = 780 nm). The lenses and pixels were fabricated by two‐photon polymerization lithography. To demonstrate SSI, we separately combined the off‐axis cylindrical Fresnel lens arrays and the encoded print to display 3 decoded images. Subsequently, we digitally reconstructed these images for visualization. The images had acceptable color contrast, as the simulated normalized modulation transfer function (*MTF*) was ≈0.6 averaged across wavelengths in the visible spectrum. This *MTF* value corresponded to the horizontal spatial frequency of the sub‐pixels in the encoded print (0.20 cycles µm^−1^), which was below the lowest cutoff spatial frequency of the lenses (0.62 cycles µm^−1^) for wavelengths in the visible spectrum.

In future works, SSI can be extended from static to dynamic sampling of pixels by designing off‐axis lens arrays with phase‐change materials or spatial light modulators. This approach will allow different images to be displayed through one actively tunable lens array, instead of mechanically switching between several off‐axis cylindrical Fresnel lens arrays with fixed phase profiles. The actively tunable lens array can also be paired with actively tunable structural color pixels in an encoded print for an extra degree of freedom. Hence, introducing tunability into the designs of the lens arrays and structural color pixels will strengthen the practical application of SSI. For example, they can be used together in an optical information security device that unlocks or grants access if the color images corresponding to the correct set of “keys” are displayed. Furthermore, 3D images can be displayed through SSI by using off‐axis lens arrays with spatially varying focal lengths, such that the different points of an image are formed on separate planes.^[^ ^38^ ^]^

## 4 Experimental Section

### Electromagnetic Wave Simulation

Pixel A and the Fresnel lenses were simulated using Ansys Lumerical 2024 Finite‐Difference Time‐Domain (FDTD) 3D Electromagnetic Solver. The pixel material and the lens material were set to “Sampled 3D data”, based on the Cauchy parameters of IP‐DIP resin exposed by two‐photon polymerization lithography (Figure S1, Supporting Information). The substrate material was set to “SiO2 (Glass)–Palik”.

- In the pixel simulation, Pixel A was modeled as a 21 × 21 array of nanopillars, with a center‐to‐center separation distance of 1 µm between adjacent nanopillars in the x and y directions. Each nanopillar comprised a cylinder (height = 1.035 µm, radius = 0.165 µm) capped with a hemisphere (radius = 0.165 µm). A broadband x‐polarized total‐field‐scattered‐field source with wavelengths ranging from 380 to 780 nm illuminated the pixel at normal incidence from the substrate side. The boundary conditions of the FDTD simulation region were set to anti‐symmetric in the x direction, symmetric in the y direction, and perfectly matched layer (PML) in the z direction. A frequency‐domain field and power (DFT) monitor placed after the nanopillars was used to calculate the electric field magnitude distributions and the transmittance spectra. The transmittance spectra were calculated for varying integration cone half angles, corresponding to numerical aperture values ranging from NA = 0.05 to NA = 0.95, in steps of 0.05. For each NA, the transmittance spectrum was normalized to a reference spectrum from a control setup (substrate without nanopillar).
- In the lens simulation, each lens was modeled as a surface defined by the corresponding thickness of the designed hyperbolic phase profile. A narrowband x‐polarized plane wave source illuminated the lens at normal incidence from the substrate side, for wavelengths = [380, 550, 780] nm. The boundary conditions of the FDTD simulation region were set to periodic in the x and y directions, PML in the z direction. A DFT monitor placed after the lens was used to calculate the electric field intensity (magnitude squared) distributions and the optical power of the focal spots.

### Raytracing Simulation

The Fresnel lenses were also simulated using MATLAB R2024a. Each lens was modeled as a surface defined by the corresponding thickness of the designed hyperbolic phase profile. Each lens was illuminated by normally incident rays for wavelengths = [380, 550, 780] nm. The lens refractive index was set according to the Cauchy parameters of IP‐DIP resin exposed by two‐photon polymerization lithography (Figure S1, Supporting Information). By applying the generalized Snell's law,^[^ ^39^ ^]^ the angles of the refracted rays were calculated.

### Nanofabrication

The Fresnel lenses and structural color pixels were fabricated on separate samples. For each sample, a drop of IP‐Dip resin was placed on a clean fused silica substrate. Each sample was then transferred to a Nanoscribe Photonic GT Professional System for two‐photon polymerization lithography. The lenses were written in continuous mode, using a laser power of 8 mW and a scan speed of 5000 µm s^−1^. The pixels were written in pulsed mode, using a laser power of 20 mW and varying exposure times per exposed voxel (0.08–0.32 ms). After writing, each sample was removed from the Nanoscribe Photonic GT Professional System. The sample comprising pixels was immersed sequentially in propylene glycol methyl ether acetate (PGMEA), isopropyl alcohol (IPA), and nonafluorobutyl methyl ether (NME). The sample comprising lenses was immersed sequentially only in PGMEA and IPA. The immersion time of each sample in each chemical solution was 300 s. Finally, the samples were dried at room temperature.

### Imaging

The optical images of the samples (Figures 2a and 4a–c) were captured in brightfield transmission mode using a Nikon LV‐HL50W halogen lamp, a Nikon T Plan SLWD 10×/0.20 objective, and a Nikon DS‐Ri2 camera. The optical images of Pixel A (Figure S4, Supporting Information) were captured using a Nikon T Plan SLWD 10×/0.20 objective, and a Nikon TU Plan 100×/0.90 objective. To prepare the samples for scanning electron microscope (SEM) imaging, the samples were sputtered using an Au:Pd 60:40 target (Ted Pella, Inc.) in a JEOL JFC‐1600 auto fine coater set at 20 mA for 150 s. The SEM images of the samples (insets of Figure 1b) were then captured by a JEOL JSM‐7600F field emission SEM system, with its acceleration voltage set to 5 kV.

### Spectra Measurement

The transmittance spectra of the structural color pixels were measured using a CRAIC Technologies 508 PV spectrometer added to a Nikon T Plan SLWD 10×/0.20 objective.

## Conflict of Interest

The authors declare no conflict of interest.

## Author Contributions

J.Y.E.C. did the ideation, simulations, experiments, and wrote the manuscript. A.R. and X.Y.L. assisted with the designs and experiments. H.W., H.T.W., and X.Y.Z. assisted with the manuscript figures and analysis of results. C.W.Q. and J.K.W.Y. supervised the research. All authors revised the manuscript.

## Supporting information



Supporting Information

## Data Availability

The data that support the findings of this study are available from the corresponding author upon reasonable request.
