# Supplementary material for: Spatially Selective Imaging in Color: What You See is What You Want
Source: Adv Sci (Weinh). 2024 Dec 27;12(7):2411537. doi: 10.1002/advs.202411537 (PMC11831530; doi:10.1002/advs.202411537)
Supplement: Supplementary file 1 — Supporting Information [file ADVS-12-2411537-s001.docx]

Supporting Information for Spatially Selective Imaging in Color: What You See is What You Want

John You En Chan^1^, Akshaya Rajesh^1^, Xiaoyu Lin^1^, Hao Wang^2^, Hongtao Wang^1^, Xiaoyan Zhou^1^, Cheng-Wei Qiu^3^, Joel K.W. Yang^1*^

^*^To whom correspondence should be addressed: joel_yang@sutd.edu.sg

^1^Engineering Product Development, Singapore University of Technology and Design, Singapore 487372

^2^School of Instrumentation and Optoelectronic Engineering, Beihang University, Beijing 100191, China

^3^Department of Electrical and Computer Engineering, National University of Singapore, Singapore 117583

**Note S1. Calculation of CIE1976 *u’*-*v’* chromaticity coordinates.**

To calculate the CIE1976 *u’*-*v’* chromaticity coordinates, the XYZ tristimulus values were first calculated using Equations S1 – S3:

| $X=\sum_{\lambda=380 \mathrm{nm}}^{\lambda=780 \mathrm{nm}} T\left( \lambda\right)\cdot S\left( \lambda\right)\cdot\bar{x}\left( \lambda\right)\cdot\Delta\lambda$ | (S1) |
| --- | --- |
| $Y=\sum_{\lambda=380 \mathrm{nm}}^{\lambda=780 \mathrm{nm}} T\left( \lambda\right)\cdot S\left( \lambda\right)\cdot\bar{y}\left( \lambda\right)\cdot\Delta\lambda$ | (S2) |
| $Z=\sum_{\lambda=380 \mathrm{nm}}^{\lambda=780 \mathrm{nm}} T\left( \lambda\right)\cdot S\left( \lambda\right)\cdot\bar{z}\left( \lambda\right)\cdot\Delta\lambda$ | (S3) |

where *T*(*λ*) is the transmittance spectrum of a pixel for a given numerical aperture (*NA)*; *S*(*λ*) is the spectral power distribution of the D65 standard illuminant; *x̄*(*λ*), *ȳ*(*λ*), *z̄*(*λ*) are the color matching functions of the CIE 1931 2° standard observer; Δ*λ* = 5 nm is the wavelength sampling interval. The tristimulus values were then converted to *u’*-*v’* coordinates using Equations S4 – S5:

| $u'=\frac{4X}{X+15Y+3Z}$ | (S4) |
| --- | --- |
| $v'=\frac{9Y}{X+15Y+3Z}$ | (S5) |

The D65 standard illuminant white point (*u_w_’*, *v_w_’*) = (0.1978, 0.4683) was calculated using Equations S1 – S5 by setting *T*(*λ*) = 1. The chromaticity difference Δ*u’v’* between the pixel and the D65 standard illuminant white point was then calculated using Equation S6:

| $\Delta u^{'}v^{'}=\sqrt{\left( u^{'}-{u_{w}}^{'} \right)^{2}+\left( v^{'}-{v_{w}}^{'} \right)^{2}}$ |  |  | (S6) |
| --- | --- | --- | --- |

| *NA* | *u’* | *v’* | *Δu’v’* |
| --- | --- | --- | --- |
| 0.05 | 0.1437 | 0.3017 | 0.1752 |
| 0.10 | 0.1441 | 0.3029 | 0.1739 |
| 0.15 | 0.1445 | 0.3041 | 0.1727 |
| 0.20 | 0.1450 | 0.3054 | 0.1713 |
| 0.25 | 0.1454 | 0.3069 | 0.1697 |
| 0.30 | 0.1458 | 0.3084 | 0.1681 |
| 0.35 | 0.1463 | 0.3102 | 0.1664 |
| 0.40 | 0.1468 | 0.3122 | 0.1642 |
| 0.45 | 0.1478 | 0.3132 | 0.1630 |
| 0.50 | 0.1428 | 0.3204 | 0.1579 |
| 0.55 | 0.1232 | 0.3951 | 0.1046 |
| 0.60 | 0.1551 | 0.4459 | 0.0482 |
| 0.65 | 0.1908 | 0.4554 | 0.0147 |
| 0.70 | 0.1960 | 0.4559 | 0.0126 |
| 0.75 | 0.1939 | 0.4579 | 0.0111 |
| 0.80 | 0.1919 | 0.4620 | 0.0087 |
| 0.85 | 0.1946 | 0.4647 | 0.0049 |
| 0.90 | 0.1982 | 0.4655 | 0.0029 |
| 0.95 | 0.1994 | 0.4656 | 0.0032 |

**Table S1.** Simulated *u’*, *v’* chromaticity coordinates of Pixel A and their chromaticity differences *Δu’v’* with the D65 standard illuminant white point, for varying numerical aperture (*NA*). The chromaticity differences were calculated using Equation S6 from Note S1.


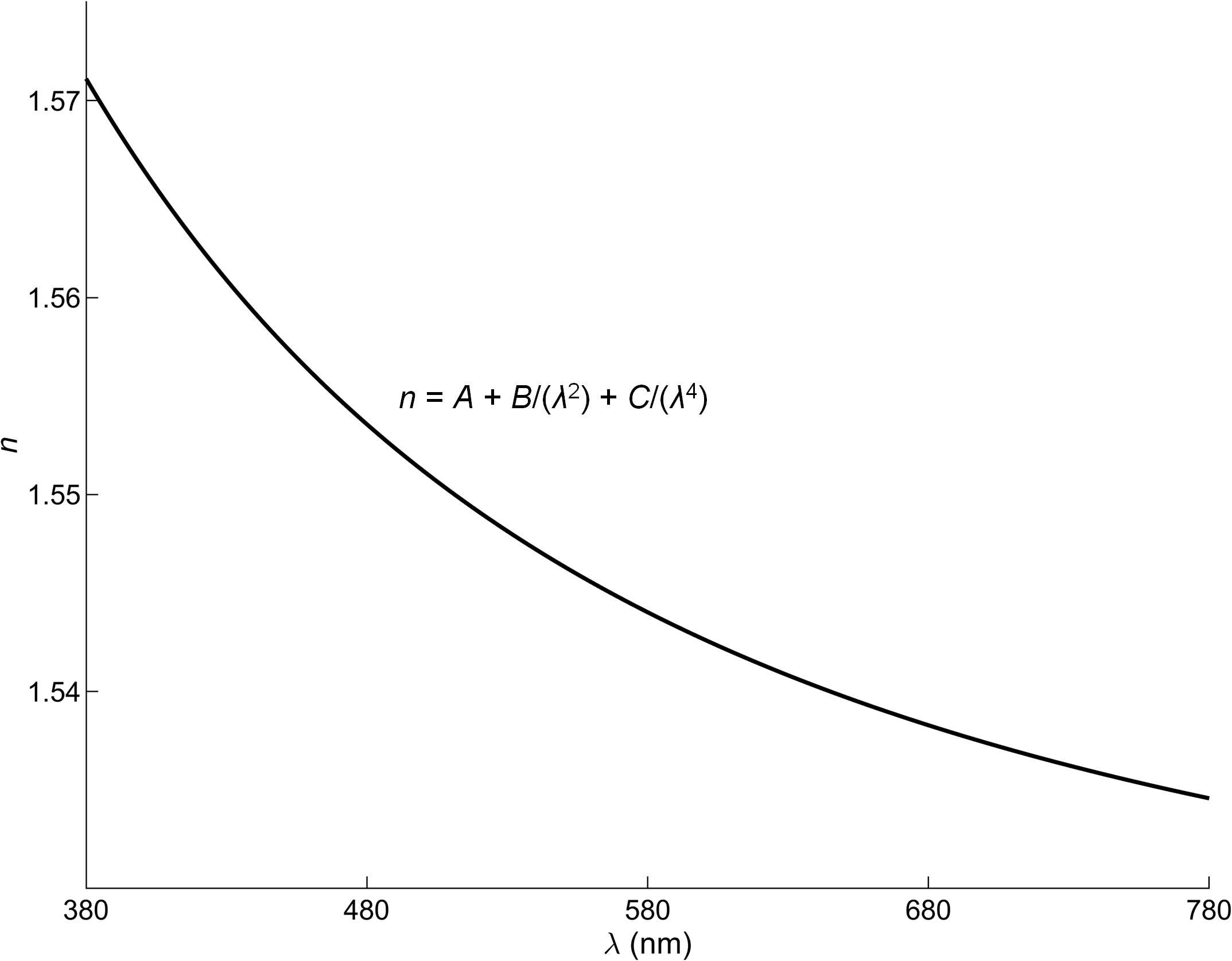


**Figure S1.** Refractive index *n* of IP-DIP resin exposed by two-photon polymerization lithography, as a function of wavelength *λ*. The Cauchy parameters are given by: *A* = 1.52266; *B* = 0.00733 µm^-2^; *C* = -0.482*10^-4^ µm^-4^.


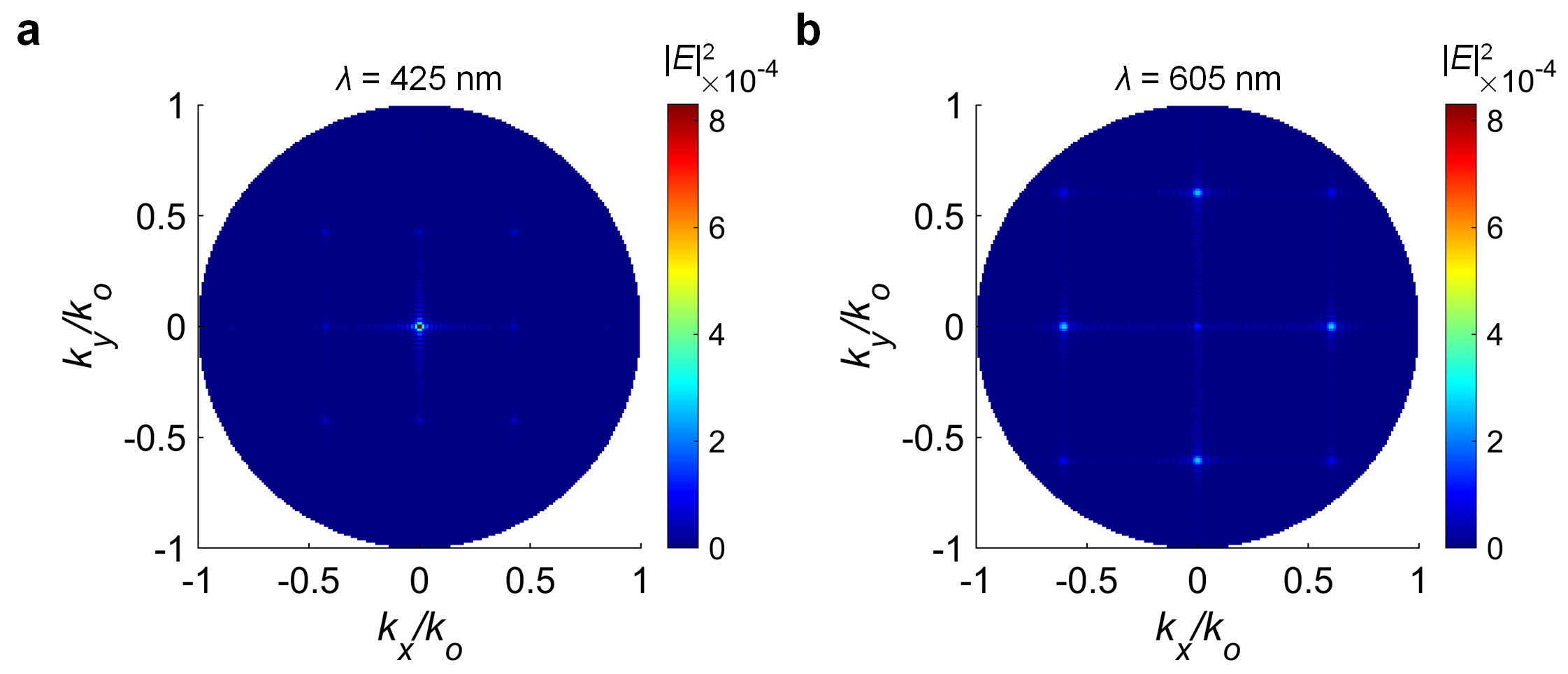


**Figure S2.** Simulated electric field intensity (|*E*|^2^) distributions in the far field for Pixel A. These distributions also represent the propagation angles of transmitted light. These distributions are shown for a) the peak wavelength *λ* = 425 nm, and b) the trough wavelength *λ* = 605 nm.


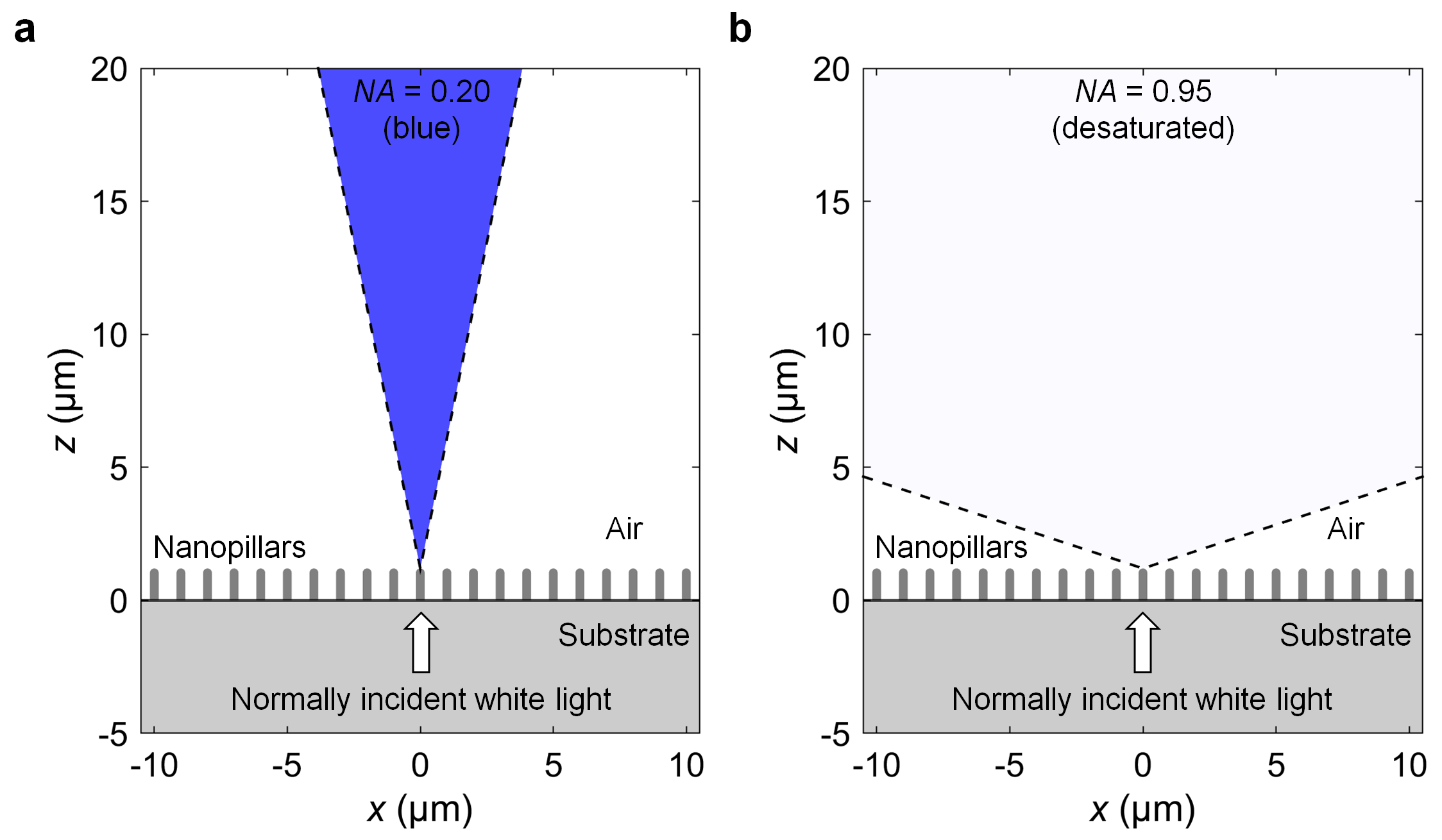


**Figure S3.** Schematics that illustrate the color dependence of Pixel A on the numerical aperture (*NA*). Pixel A is represented by the array of nanopillars. a) *NA* = 0.20 is represented by the blue region, whereas b) *NA* = 0.95 is represented by the desaturated region.


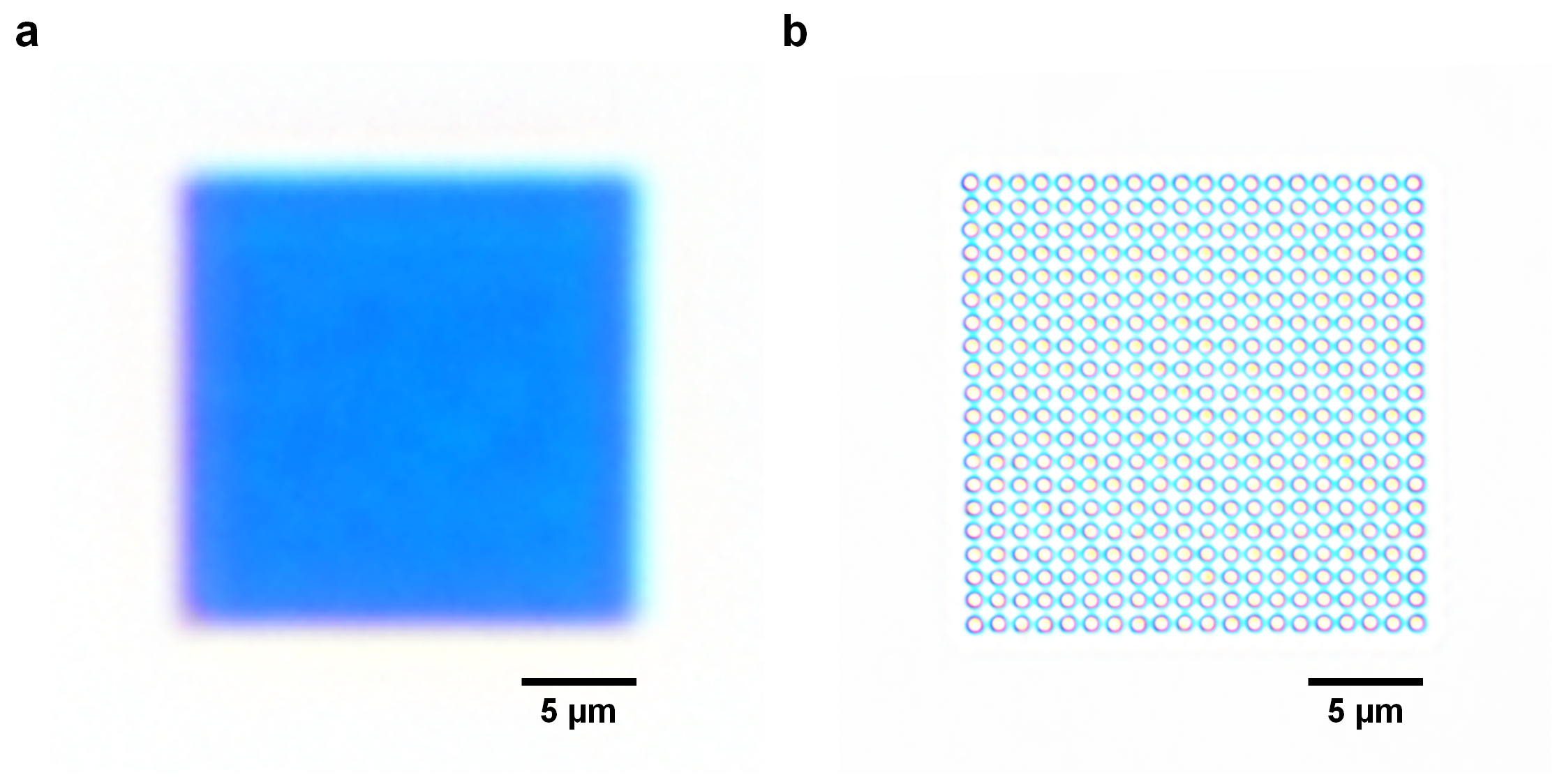


**Figure S4**. Optical images of Pixel A captured using microscope objectives with different numerical aperture (*NA*). a) Image captured using a 10× objective with *NA* = 0.20. b) Image captured using a 100× objective with *NA* = 0.90.


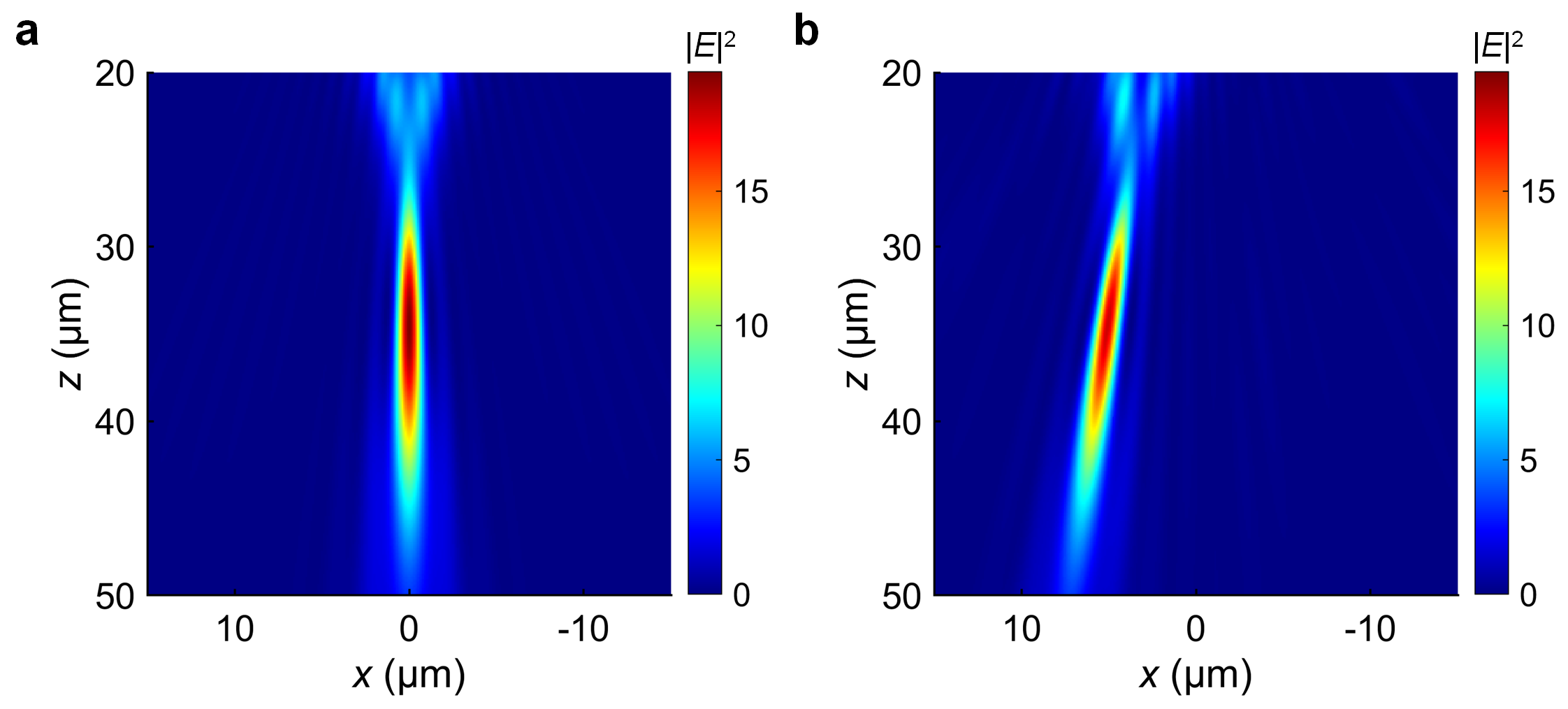


**Figure S5.** Simulated electric field intensity (|*E*|^2^) distributions of the focal spot in the x-z plane at y = 0 µm for wavelength *λ* = 550 nm. The distributions are shown for a) the on-axis lens, and b) the off-axis lens.


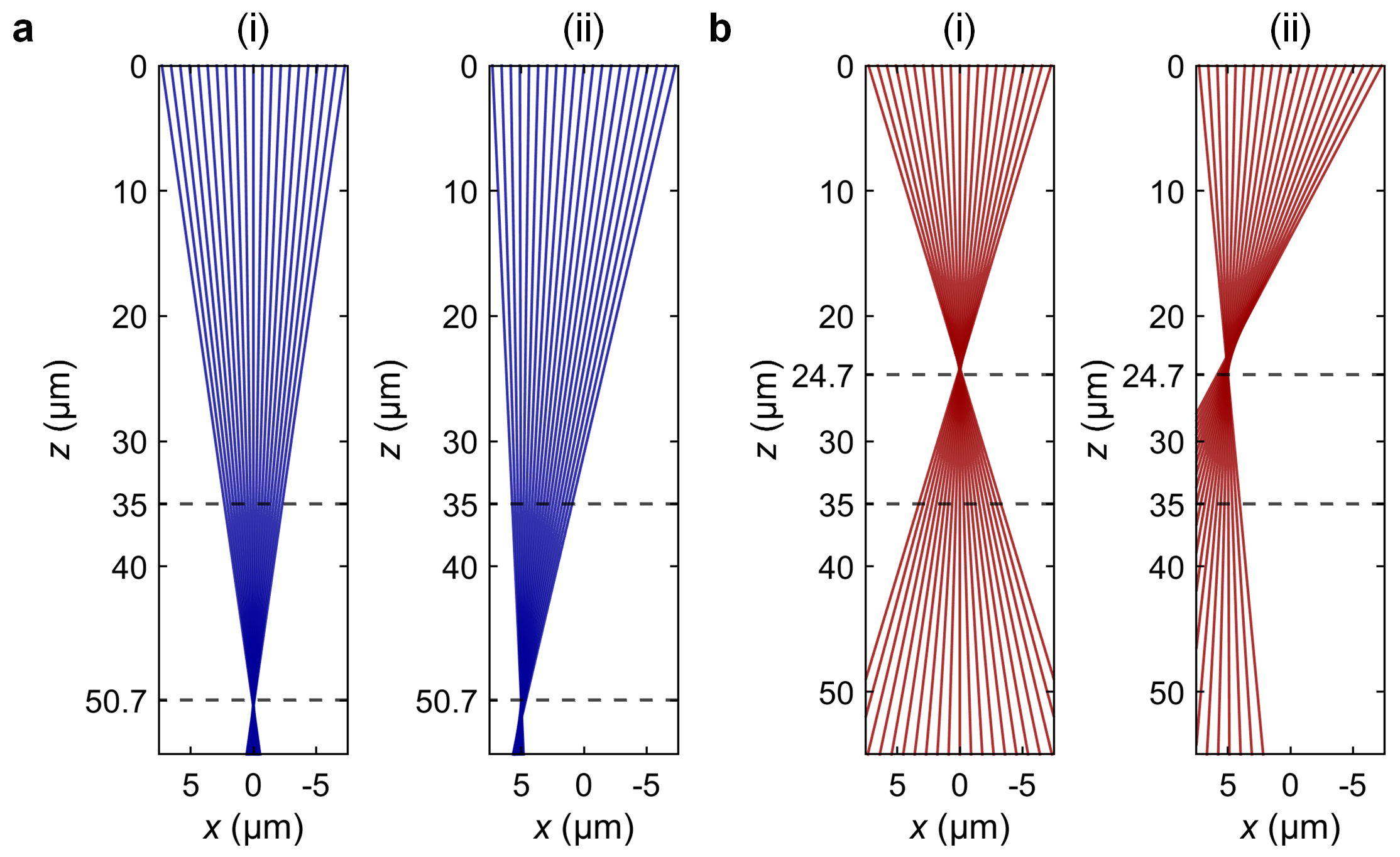


**Figure S6.** Raytracing diagrams of the designed lenses for wavelengths *λ* at the limits of visible spectrum. The diagrams are shown for a) *λ* = 380 nm and b) *λ* = 780 nm, in (i) the on-axis lens and (ii) the off-axis lens.


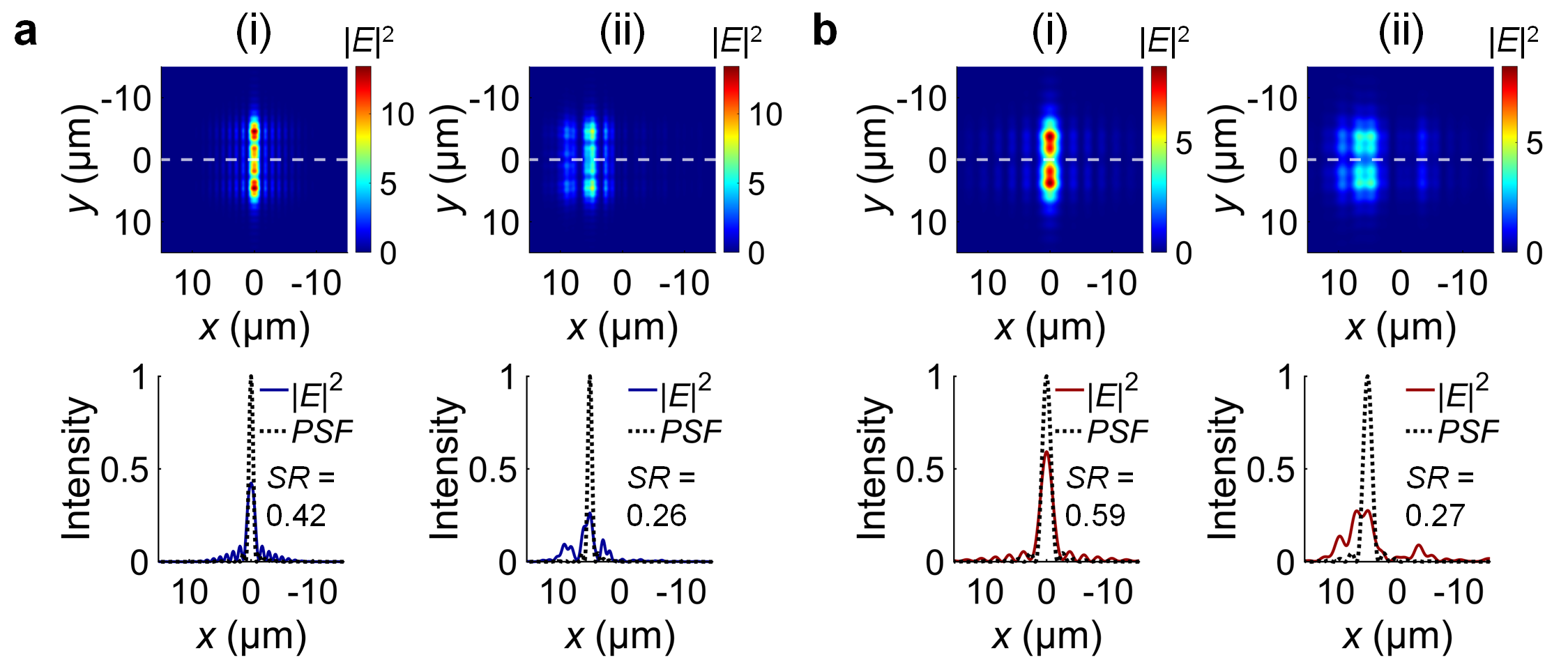


**Figure S7.** Simulated electric field intensity (|*E*|^2^) distributions and normalized profiles of the Fresnel lenses for wavelengths *λ* at the limits of visible spectrum. The electric field intensity distributions were simulated on the *x*-*y* plane at *z* = 35 µm, and the electric field intensity profiles were examined along the *x*-direction at *y* = 0 µm (white dashed line). Each profile was normalized to yield the same area under the curve as the point spread function (*PSF*) for that lens and wavelength. The distributions and profiles are shown for a) *λ* = 380 nm and b) *λ* = 780 nm, in (i) the on-axis lens and (ii) the off-axis lens. *SR* refers to the Strehl ratio.


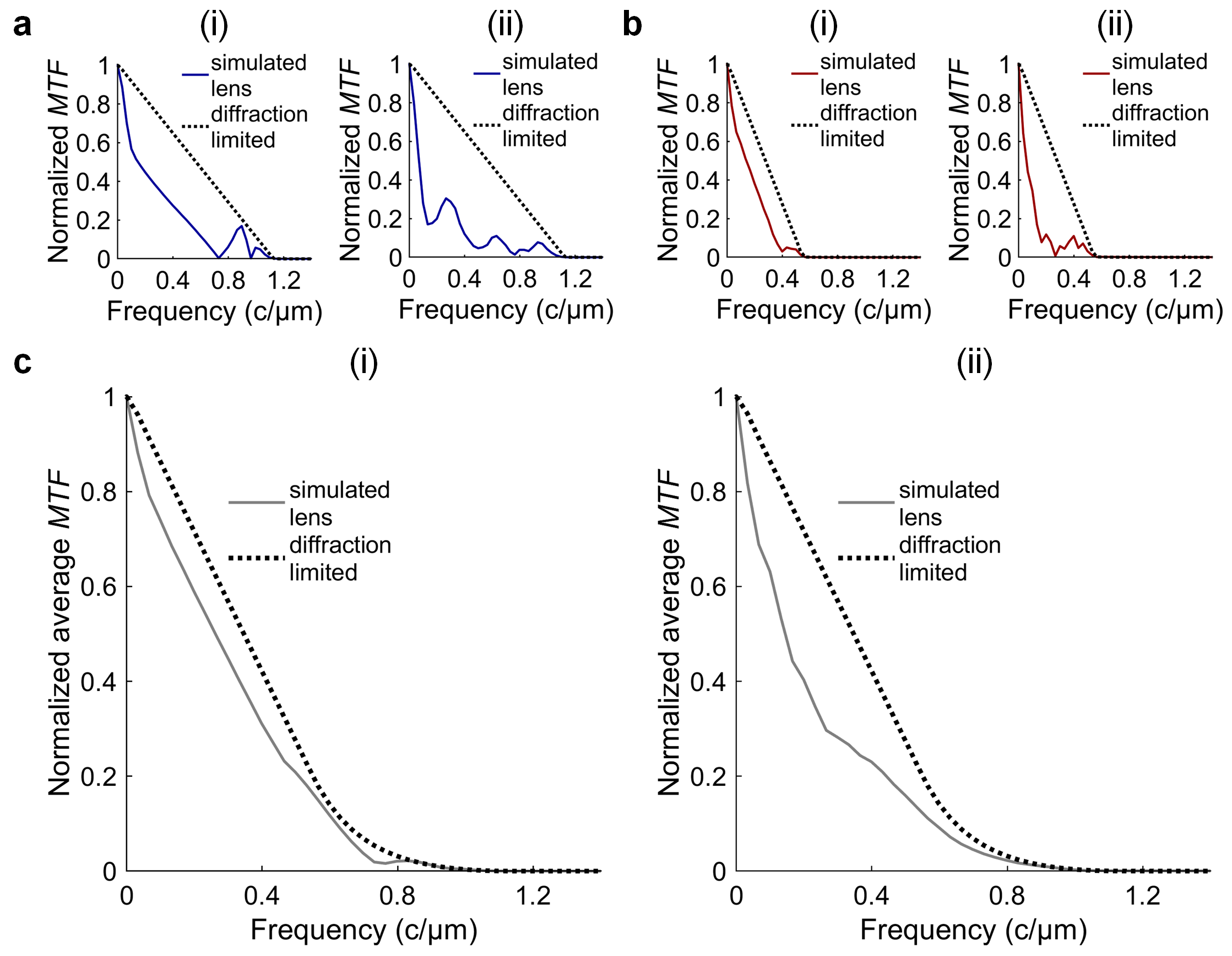


**Figure S8.** Normalized modulation transfer function (*MTF*) graphs of the simulated lenses and their diffraction-limited cases for wavelengths *λ* across the visible spectrum. The simulated lenses are (i) the on-axis lens, and (ii) the off-axis lens. a) Normalized *MTF* graphs for *λ* = 380 nm. b) Normalized *MTF* graphs for *λ* = 780 nm. c) Normalized average *MTF* graphs. The average *MTF* was calculated by the equally weighted sum of *MTF* from *λ* = 380 nm to *λ* = 780 nm in constant step of 5 nm, divided by the total number of wavelength samples. The average *MTF* was then normalized to yield a value of 1 at zero spatial frequency.


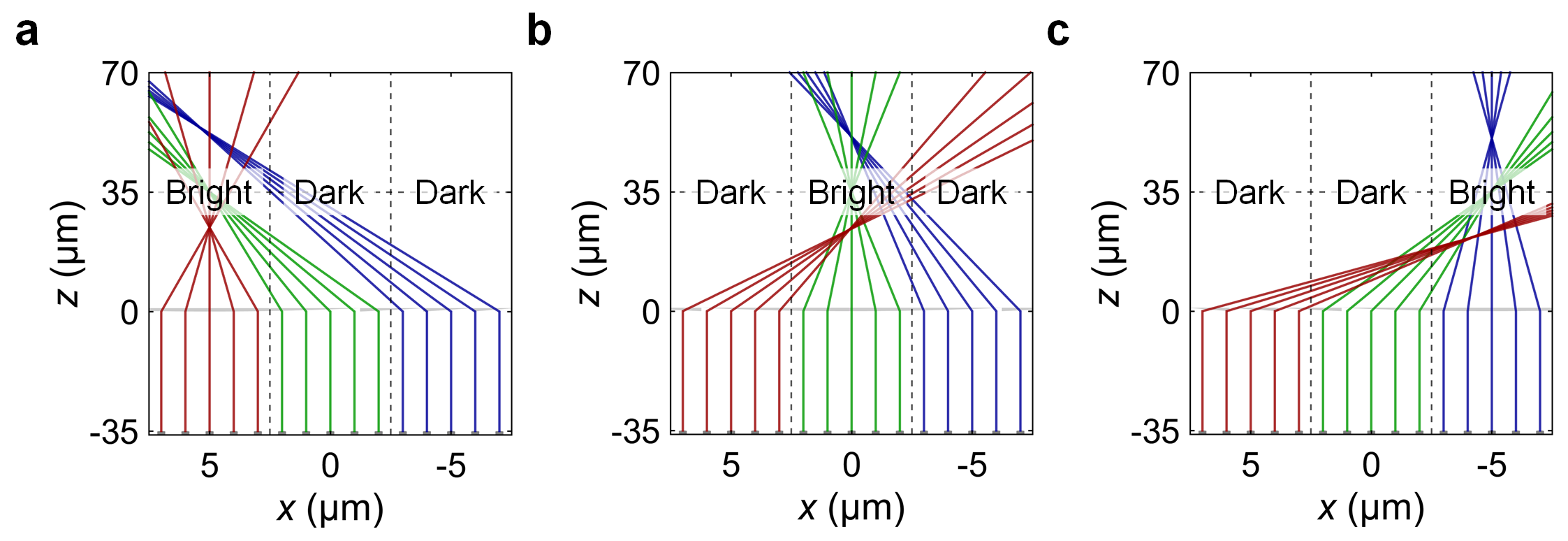


**Figure S9.** Simulated raytracing diagrams that show how each lens focuses transmitted light from three different pixels. The lens is at the plane *z* ~= 0 µm, and the pixels are at the plane *z* ~= -35 µm. For simplicity, each pixel is assumed to transmit only a specific wavelength of light that propagates parallel to the *z*-axis. The wavelengths are assumed to be *λ* = 780 nm (red), *λ* = 550 nm (green), and *λ* = 380 nm (blue). The focal spot image of each lens is captured at the plane *z* = 35 µm, where the focal spot appears in the bright area, but not in the dark area. The raytracing diagrams are shown for the designed lenses: a) *x_F_* = 5 µm, b) *x_F_* = 0 µm, and c) *x_F_* = -5 µm, in which *x_F_* denotes the *x*-position of the focal point.
